# Difficulties experienced by veterinarians when communicating about emerging zoonotic risks with animal owners: the case of Hendra virus

**DOI:** 10.1186/s12917-017-0970-2

**Published:** 2017-02-18

**Authors:** Diana H Mendez, Petra Büttner, Jenny Kelly, Madeleine Nowak, Rick Speare (posthumously)

**Affiliations:** 1 College of Public Health, Medical and Veterinary Sciences, James Cook University, Townsville, QLD 4811 Australia; 2 Centre for Chronic Disease Prevention, James Cook University, Cairns, QLD 4870 Australia; 3 Tropical Health Solutions Pty Ltd, Townsville, 4811 QLD Australia; 4 College of Medicine and Dentistry, James Cook University, Townsville, QLD 4811 Australia; 5 Centre for Nursing and Midwifery Research, James Cook University, Townsville, QLD 4811 Australia

**Keywords:** Veterinarians, Horse owners, Emerging zoonoses, Hendra virus, Risk communication, Health and safety

## Abstract

**Background:**

Communication skills are essential for veterinarians who need to discuss animal health related matters with their clients. When dealing with an emerging zoonosis, such as Hendra virus (HeV), veterinarians also have a legal responsibility to inform their clients about the associated risks to human health. Here we report on part of a mixed methods study that examined the preparedness of, and difficulties experienced by, veterinarians communicating about HeV-related risks with their clients.

**Methods:**

Phase 1 was an exploratory, qualitative study that consisted of a series of face-to-face, semi-structured interviews with veterinary personnel from Queensland, Australia (2009–10) to identify the barriers to HeV management in equine practices. Phase 2a was a quantitative study that surveyed veterinarians from the same region (2011) and explored the veterinarians’ preparedness and willingness to communicate about HeV-related risks, and the reactions of their clients that they experienced. The second study included both multiple choice and open-ended questions.

**Results:**

The majority of the participants from Phase 2a (83.1%) declared they had access to a HeV management plan and over half (58.6%) had ready-to-use HeV information available for clients within their practice. Most (87%) reported “always or sometimes” informing clients about HeV-related risks when a horse appeared sick. When HeV was suspected, 58.1% of participants reported their clients were receptive to their safety directives and 24.9% of clients were either initially unreceptive, overwhelmed by fear, or in denial of the associated risks. The thematic analysis of the qualitative data from Phases 1 and 2a uncovered similar themes in relation to HeV-related communication issues experienced by veterinarians: “*clients’ intent to adhere*”; “*adherence deemed redundant*”; “*misunderstanding or denial of risk*”; “*cost*”; “*rural culture*”; “*fear for reputation*”. The theme of “*emotional state of clients*” was only identified during Phase 1.

**Conclusion:**

Warning horse owners about health and safety issues that may affect them when present in a veterinary work environment is a legal requirement for veterinarians. However, emerging zoonoses are unpredictable events that may require a different communication approach. Future training programs addressing veterinary communication skills should take into account the particular issues inherent to managing an emerging zoonosis and emphasise the importance of maintaining human safety. Veterinary communication skills and approaches required when dealing with emerging zoonoses should be further investigated.

**Electronic supplementary material:**

The online version of this article (doi:10.1186/s12917-017-0970-2) contains supplementary material, which is available to authorized users.

## Background

Veterinarians routinely communicate with their clients about a range of topics including animal health, wellbeing and lifestyle issues, animal-human interactions, animal behaviour, end-of-life decisions and cost for services provided [1]. However, some veterinarians have reported feeling uncomfortable communicating about some of these topics: diagnosis, treatment options (including euthanasia) and cost. [2–5] For example, some veterinarians found the conversation about veterinary fees difficult due to apprehension about clients’ reactions, which may range from upfront acceptance; to suspicion; to disagreement or refusal to comply with a treatment plan; to a client not returning to the practice [6–8]. Communication between veterinarians and their clients can by affected by a number of factors: 1) communication skills of veterinarians; 2) misalignment between veterinarians’ and clients’ motivations 3) type of situation: “routine” vs “crisis” ; 4) perceptions of clients about the role of veterinarians; 4) veterinarian’s ability to communicate with the whole spectrum of clients, and 5) clients’ ability to understand the message [3–5, 8–26]. However, clients’ adherence with veterinary recommendations and directives has been shown to improve with a “client-centred” communication approach [27].

Breakdown in communication between veterinarians and their clients may affect animal health outcomes and the level of client satisfaction [2]. Poor communication may also have serious occupational health and safety repercussions for both veterinary personnel and animal owners. In Australia, for example, veterinarians have a legal responsibility to prevent their staff and clients from becoming exposed to chemical, physical or infectious risks in the veterinary work environment [28]. This is particularly relevant when managing zoonoses, infectious diseases transmissible from vertebrate animals to humans. Veterinary mitigation of known zoonoses has been reported as being less than adequate; leading to occupational infection of veterinary personnel, veterinary students and animal owners [29–34].

The relatively recent emergence of Hendra virus (HeV) in Australia has further highlighted these veterinary deficiencies and the challenges faced by veterinarians when managing a previously unknown zoonosis [35]. Since 1994, HeV has spilled-over from flying foxes into horses on at least 52 occasions and in seven instances into humans: four veterinary personnel (two died, two survived); a horse trainer (died); a stable hand (survived) and a horse owner (died) [35, 36]. Transmission from horses to humans is via exposure to bodily fluids, including blood, from an infected horse which may have been shedding viral HeV particles 2 days prior to developing clinical signs [35–37]. Although infection is rare, the mortality rate is 57% in humans, hence its health and safety significance for veterinarians and horse owners [35, 36].

When a horse is suspected of HeV, veterinarians are mandated to notify the biosecurity authorities; and while awaiting field support from these authorities, they need to inform horse owners about the risks involved and, if necessary, instruct them about infection control (IC) strategies to mitigate these risks [38]. Failing to do so would have legal repercussions for veterinarians and their practices [28, 39]. Hence, from a health and safety, legal and business perspective, it is important veterinarians are able to communicate with clients effectively about zoonotic risks.

**Fig. 1:**
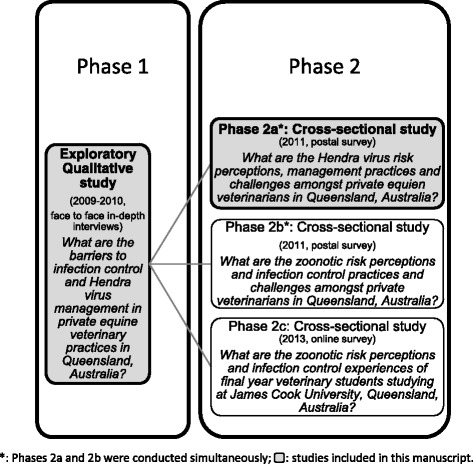
Understanding infection control implementation and Hendra virus management in private veterinary practices: mixed methodology diagram

As part of a larger mixed methods sequential exploratory study, which aimed to identify and understand the factors affecting veterinary IC and HeV management in private veterinary practices (Fig. 1), HeV-related risk communication issues were identified and further investigated [40–42]. The work presented here only focuses on the parts of the study that examined the relevant risk communication issues and the possible effect these may have on the management of HeV.

## Methods

### Research design

A mixed methods exploratory sequential design was used (Fig. 1) [43]. The aim of Phase 1, a qualitative exploratory study, was to identify the barriers to, and enablers of, HeV management in private veterinary practices in Queensland (Qld), Australia (Fig. 1) [40, 41]. Phase 2 of the study consisted of three cross-sectional studies (Phases 2a, 2b, 2c) (Fig. 1) which sought to further examine the barriers to HeV management and veterinary IC identified during Phase 1 [42]. The design of the survey tools used in the second phase of the study was informed by the results obtained during Phase 1 of the study [40, 41]. The first cross-sectional study (Phase 2a) focused on the issues surrounding HeV management, including risk communication between veterinarians and horse owners [42]. Only the part relevant to HeV-related risk communication with clients from Phase 2a is presented here. The research designs and protocols and some results from Phases 1 and 2 have been published elsewhere [40–42]. The qualitative components of the overall study complied with the qualitative research guidelines on relevance, appropriateness, transparency and soundness of research methodology (RATS) [44, 45].

#### Phase 1

Phase 1 consisted of a series of 21 semi-structured, face-to-face in-depth interviews with veterinary personnel from 14 private practices providing equine services along the eastern coast of Qld, Australia, between December 2009 and September 2010 [40, 41]. The purposively recruited participants included thirteen males and eight females between the ages of 28 and 63 years. Eight were veterinarian employees, ten were principal veterinarians who estimated spending between 2 and 100% of their time working with equine patients. Two veterinary nurses who worked regularly with equine patients and one practice manager were also interviewed. Participants were all recruited within the known distribution range of HeV [40, 41]. Nine worked in practices located in areas classified as metropolitan, nine in rural areas and three were remotely located [40, 41]. The questions included in the interview guide were developed after consultation with a reference group which included private veterinarians, biosecurity and health and safety government representatives, members of the Australian Veterinary Association and Equine Veterinarians Australia, and public health and veterinary academics (Additional file 1). The open-ended questions explored the participants’ experiences and views about the barriers to and enablers of HeV management and related IC practices. Participants were not asked specifically about possible communication issues, but most participants spontaneously broached the subject during the interviews.

#### Phase 2a

During Phase 1, veterinarians reported having experienced various degrees of difficulty communicating HeV-related risk information and risk mitigation instructions to clients who, in some instances, were akin to lay assistants [41]. Following these findings, communication issues between veterinarians and their clients were further investigated during Phase 2a, along with other issues identified during Phase 1 [42]. Phase 2a consisted of a cross-sectional postal survey of all veterinarians working in private practice in Qld between June 2011 and September 2011 [42]. However, only those who had provided veterinary services to a horse patient at least once in the 12 months prior to taking part in the survey were eligible to participate in the study. Aside from a number of socio-demographic, education and professional questions, participants were asked: 1) if they had access to a formally documented HeV management plan within their practice; 2) if the HeV management plan contained information for horse owners about HeV and flying foxes; 3) how often they provided HeV information and safety instructions according to the health status of a horse (healthy or sick); and 4) how often they instructed a lay assistant about the potential risk of exposure to HeV depending on the health status of the horse (Additional file 2). Frequency of HeV communication with clients was recorded as “always”, “sometimes”, “rarely”, “never”. Participants were also asked open-ended questions about the reactions of lay assistants/horse owners in regards to HeV risk communication and safety instructions when HeV was suspected. The survey was pilot-tested with three veterinarians from the target population who provided feedback on wording, format, and appropriateness of questions. Very few, and only minor, modifications were made.

### Data analysis

#### Phase 1

Each participant was assigned an individual and a practice alphanumeric identifier (V* for veterinarians, VN* for veterinary nurses, PM* for practice manager, P* for practice). The iterative thematic analysis of the qualitative data collected during Phase 1 yielded six main themes, including “*risk and risk mitigation communication*” [41]. The data coded to this particular theme were subsequently reanalysed thematically for further units of meaning in order to better conceptualise the communication issues between veterinarians and their clients. This process was repeated on several occasions by the first author (DM) and outcomes were reviewed by the last author (RS) for congruence and meaningfulness within the context of the management of HeV in private veterinary practices.

#### Phase 2a

Completed and returned questionnaires were de-identified and attributed a unique alphanumeric identification, P*. Responses were collated into Excel (Microsoft. Released 2010) and later imported into SPSS (IBM Corp. Released 2012. IBM SPPS Statistics for Windows, Version 21.0. Armonk, NY: IBM Corp.). Categorical descriptive data about HeV management, related risk and risk mitigation communication issues, and socio-demographic information were reported using percentages. The responses about HeV-related risks communication were collapsed into two categories: “always or sometimes” and “rarely or never”. Data collected from open-ended responses to questions about the reaction of horse owners/lay assistants to HeV-related risk warnings and safety instructions were categorised into: “*lay assistant was receptive and complied*”; “*lay assistant was unreceptive but complied*”; “*lay assistant was in denial*”; “*lay assistant was overcome with fear*”; “*no lay assistant was used*”; and “*lay assistant had another type of reaction not previously described*”, then quantified and reported using percentages (Table 1). In order to further identify and understand the barriers to communication of HeV-related risk and risk mitigation with clients, the data from the open-ended questions were further analysed thematically by both DM and RS following the same procedure as in Phase 1. The resulting themes were subsequently triangulated with the themes obtained during Phase 1 about the issues surrounding risk communication between veterinarians and their clients.

**Table 1:** Access to HeV management plan and HeV-related risk and safety communication to horse owners and/or lay assistants and their reactions according to 200* participants during the winter of 2011. (Phase 2a)

Characteristics	Frequencies (relative frequencies)
*HeV management plan in the practice* (*n* = 195)	
Yes	162 (83.1%)
No	27 (13.8%)
Don’t know	6 (3.1%)
*Are the following information documents available in the practice*	
Information sheet about HeV for horse owners (*n* = 191)	112 (58.6%)
Information sheet about flying foxes for horse owners (*n* = 191)	61 (31.9%)
*Provision of HeV risk related education to horse owners if horse is healthy* (*n* = 189)	
Always or sometimes	88 (46.6%)
Rarely or never	101 (53.5%)
*Provision of HeV risk related education to horse owners if horse is sick* (*n* = 191)	
Always or sometimes	166 (87%)
Rarely or never	25 (13.1%)
*How often did veterinarians instruct lay assistants of potential risk of exposure to HeV if horse was healthy* (*n* = 186)	
Always or sometimes	93 (50%)
Rarely or never	93 (50%)
*How often did veterinarians instruct lay assistant of potential risk of exposure to HeV if horse was sick* (*n* = 189)	
Always or sometimes	172 (91%)
Rarely or never	17 (8.9%)
*Lay assistant’s reaction to risk communication about HeV (n = 133)*	
Receptive comply	55 (41.4%)
Unreceptive but comply	6 (4.5%)
Denial	15 (11.3%)
Fear	26 (19.5%)
No lay assistant used	5 (3.8%)
Other	26 (19.5%)
*Lay assistant’s reaction to safety instructions when HeV suspected* (*n* = 124)	
Receptive/comply	72 (58.1%)
Unreceptive but comply	4 (3.2%)
Denial	16 (12%)
Fear	12 (9.7%)
Never had to or help not used	10 (8.1%)
Other	10 (8.1%)

*n = 200 unless otherwise stated

Both studies received approval from the James Cook University Human Ethics Committee (approvals no.H3513, no.H3687).

## Results

### Phase 1

All of the veterinary personnel interviewed were aware of their legal responsibilities in regards to HeV-related risks to human health and had some level of HeV management plan in place. The analysis of the data coded to the theme “*Risk and risk mitigation communication*” yielded a further seven subthemes relating to the reported reactions of horse owners to veterinary advice and instructions about IC and HeV management: 1) “*Clients’ intent to adhere*”; 2)“*Adherence deemed redundant*”; 3) “*Misunderstanding or denial of risk*”; 4) “*Cost*”; 5) “*Rural culture*”; 6) “*Fear for reputation*”; and 7) “*Emotional state of clients*”.

### Phase 2a

A total of 1604 potentially eligible veterinarians were sent a survey; 200 veterinarians returned their questionnaire [42]. Not all participants answered all questions. A response rate could not be calculated because the denominator (the total number of veterinarians who had treated a horse in the past 12 months) was unknown.

The socio-demographic, professional, educational and practice profile of participants have been presented elsewhere [42]. The majority of respondents were female (52%), aged 40 years of age or older (58%), who had graduated from a Qld university (78%) and worked in a practice located in a highly to moderately accessible area (63%) as defined by the Accessibility/Remoteness Index of Australia [46]. Over half of the participants were employees (51.8%), worked full-time (89.5%) in mixed practice (79.3%) and the majority had access to a formalised HeV management plan (83.1%) [40]. Most of them reported providing veterinary services to equine patients at least weekly (77.4%) [42]. Two thirds had dealt with at least one potential case of HeV (66%) and 61.5% had attended an IC-HeV management training workshop in the previous 12 months [42].

More than half the participants reported their HeV management plan included an information sheet about HeV for horse owners (58.6%) (Table 1). Less than half the participants (46.6%) “*always or sometimes”* provided HeV-related education to horse owners when a horse appeared healthy while most (87%) did so when a horse appeared sick (Table 1). The proportion of participants who reported “*always or sometimes*” instructing lay assistants about the potential risk of exposure to HeV according to apparent health status of horse were similar to those of participants providing HeV education to horse owners (Table 1).

Of the 48 participants who answered the open-ended questions, 41.4% reported lay assistants/horse owners usually were receptive to HeV-related information and willing to adhere with safety instructions, 19.5% reported lay people were often overcome by fear and 11.3% considered they were in denial about the information provided (Table 1). The number of participants (58.1%) reporting lay assistants being receptive and willing to adhere with the safety instructions increased when a horse was suspected of being infected with HeV (Table 1).

The thematic analysis from Phase 2a yielded similar themes to those relating to risk communication identified during Phase 1, with some variations within subthemes. One of the sub-themes identified during Phase 1, “*emotional state of clients*”, did not recur in Phase 2a.

### Phases 1 and 2a: triangulation of qualitative data

Each of the themes below are illustrated by selected quotes from participants’ responses collected during Phases 1 and 2a.

#### Clients’ intent to adhere

Overall HeV-related risk and risk mitigation communication to clients was perceived as a significant issue interfering with veterinarians’ compliance with their animal welfare and occupational health and safety legal responsibilities. One participant from Phase 1 summed up the issue as illustrated in the following quote:

“*I find great difficulty in dealing with owners because it is a power play, and ultimately we are responsible of the safety of all involved. But some owners don’t believe that, which compromises the legal situation. We usually end up taking the risk out of concern for the welfare and wellbeing of the horse*.”(V4/Pc)

Other participants from Phases 1 and 2a did not find this aspect of HeV management a major challenge. Those who were successful in liaising with clients about HeV issues usually conveyed their expert knowledge about the risks involved confidently. One participant from Phase 1 reported:

“*I have not had problems with owners complying* … *You just need to make them aware of the situation and the risks involved*.” (V17/Pm)

While a participant from Phase 2a remarked:

“[Horse owners] *comply as they have been informed and educated about the risks.”* (P3553)

### Adherence deemed redundant

Although, a number of participants from Phases 1 and 2a declared that some clients appreciated being warned about the health risks posed by HeV to humans and animals and instructed on ways to manage these risks; they also pointed out that some horse owners did not see the need to use personal protective equipment (PPE). Participants further explained that horse owners, who had already been in prolonged close contact with their sick animal, believed they had already been significantly exposed to the potential risks and did not require PPE. For example the following participants highlighted:

“*The owner had already spent half a day with the sick horse, so he declined the mask because* [he] *thought exposure had already happened*.”(V7/Pa)

“*Most clients refuse PPE as they have already been handling the horse, so they don’t feel it necessary to use PPE.”*(P2444)

Veterinarians identified this as a major issue because if a client became infected it would be impossible to determine when infective exposure had occurred: before or after the involvement of the veterinarian. A participant from Phase 2a reported that:

“[Horse owners] *usually have been in very close contact with horse. So in theory, they have been exposed before vet has examined/tested the horse*.”(P3221)

#### Misunderstanding or denial of risk

Some veterinarians reported that horse owners disregarded the information they provided about HeV management. Participants thought this was because horse owners either failed to recognise the expertise of the veterinarian, the seriousness of the associated risks or were unable to follow the health and safety instructions provided. For example, participants explained:

“*You have to protect yourself first and foremost, but owners don’t see that way*.”(V8/Pe)

“*People don’t listen*.”(V14/Pj)

“*Usually* [horse owners] *make mistakes despite explicit instructions*.”(P4002)

“*They are in denial and think you are overreacting*.”(P3472)

Participants further explained that clients often did not have enough knowledge about the risks involved and/or did not have a sufficient level of understanding of biosecurity principles to follow their instructions. One participant reported:

“*Lay assistants are often reluctant to consider the disease. A lot of people don’t know anything about it*.”(P2097)

While another revealed:

“*It is difficult to explain biosecurity to people with no prior* [understanding of the] *concept*.”(P4002)

Some risk and risk mitigation communication issues encountered by veterinarians were similar to those they faced during Equine influenza outbreaks; indicating that communication challenges were not specific to the management of HeV. One participant from Phase 1 explained that:

“*I had one horse who came down with EI* [Equine influenza] *… and the next thing the others* [horses] *did too because the owners did not listen to what you said*.”(V14/Pj)

### Cost

Veterinarians interviewed, as well as those surveyed, reported feeling pressured by clients into focusing on cost minimisation rather than human health and safety. Any extra cost incurred by the management of a suspected case of HeV was said to require a justification to the client. This point is clearly articulated in the following quotes:

“*Sometimes owners put too much pressure* … *not to use infection control to lower cost, but it increases the risks*.”(V1/Pa)

“*Cost is an issue* … *What if the case turns out to be negative? How do you justify it* [cost]*?*”(V10/Pg)

“*Clients will not accept extra cost* … *for what they consider to be unnecessary PPE*.”(P2925)

Some participants further explained that according to some clients HeV testing should be done at the veterinarians’ expense. One interviewee speculated that the issue surrounding cost probably led to some HeV cases not being reported:

“[Some say] *if you want to test the horse why don’t you pay for it*! … *There would be cases out there that have not been reported because of the cost*.”(V14/Pj)

#### Rural culture

Some participants felt that their rural clientele was less receptive and accepting of their veterinary expertise or of the cost incurred by the management of a HeV case, than their urban counterparts. A number of veterinarians interviewed reported that their recommendations had been disregarded because clients preferred to rely on local traditional knowledge and past personal experiences rather than on the educated professional advice of a veterinarian. Cost was also perceived as more common issue in rural areas. The following participants recounted:

“*We were following biosecurity measures* … *people were driving in out of properties with the attitude of: “She’ll be right* [it’s alright]*, I won’t pet the horses*.”(V7/Pa)

“*My typical rural horse owner refuses to accept extra cost for exams and treatment of mildly ill horse*.”(P2227)

In some instances the use of PPE was seen by rural clients as a sign of weakness rather than a sign of professional and responsible competence. Thus some veterinarians were reluctant to follow best occupational risk mitigation strategies as they felt too self-conscious to use PPE in rural settings. These participants reported:

“*In more rural situations you tend to think you are being a bit of a Wally* [silly] *dressed up* [in PPE] *for minor issues*.”(V10/Pg)

“[In rural settings] *The cautious vet will often end up looking like it was a stupid fuss about nothing*.”(P2227)

#### Fear for reputation

Participants reported that some clients were wary of having a declared suspected case of HeV on their property because of the possible detrimental repercussions to their business and reputation. For example, one participant from Phase 2a claimed:

“*Owners generally become nervous. They worry about gossip spreading about potential HeV cases and possible media involvement*.”(P2717)

While a participant from Phase 1 added that this fear was possibly another cause for underreporting of potential HeV cases:

“*I am worried owners might not notify of infectious diseases* [including HeV] *occurrences because don’t want to be in the public eye*.”(V1/Pa)

Other interviewees made a distinction between clients who owned horses as part of their professional activity and those who owned horses for recreational purposes, explaining that a potential HeV outbreak had a different significance for different types of owners. Those working in the horse industry were perceived by participants as having more to lose from an outbreak of HeV than other types of horse owners: loss of animal assets, loss of access to animals (quarantine), loss of income. The quote from the following participant illustrates this point well:

“*The racehorse trainers don’t like to see a vet in a suit* [PPE]. *They tend to panic. If a horse suddenly dies in a riding school, they are obviously very nervous.* … [They] *worry their horses may be put in quarantine. It’s a big threat to them*.”(V18/Pn)

#### Emotional state of clients

Many professionals from the horse industry reportedly worried about the consequences for their businesses of investigation for HeV. Meanwhile participants, from Phase 1, reported many clients who owned horses for recreational purposes had difficulties accepting veterinary recommendations and became very emotional about the situation. Some of these emotions seemed to be related to their concern for their animals. Some owners disregarded veterinary safety instructions in order to stay close to their animals, or refused testing of their animals for fear of mandatory euthanasia if found infected with HeV. Participants explained:

“*The owners* … *refused testing because if* … *positive for HeV they would have to euthanise the horse. They considered their horses like their children and said: “I wouldn’t euthanise my own child”*.”(V9/Pf)

“*There was a family* … *all sobbing and cradling the horse’s head. No matter what I said they weren’t listening. Their emotions overrode their ability to follow my instructions.*”(V14/Pj)

According to some other veterinary personnel interviewed, health and safety and quarantine instructions were sometimes misconstrued as lack of empathy and care. A veterinary nurse observed:

“*Clients sometimes think that vets and nurses don’t care. They don’t understand when you tell them they cannot comfort their horse, they cannot approach their horse or bury it where they want*.”(VN2/Pe)

The impetus to follow strict HeV management measures, despite clients’ request to continue accessing their animal, had resulted in some veterinary practices losing clientele, business and reputation as one participant recounted:

“*The vet* … *was worried it may have been Hendra.* [He] *treated the horse for its clinical signs*, sent the samples off and as usual you get into the frustration of waiting for results. … *The clients were phoning us and we’d have to say no we really don’t want you to go near the horse. We told the client we can come out and give the horse more pain relief but that is it. They then phoned another practice. We sent the people a bill and they then wrote us a letter telling us how horrible we were and how distressed their daughter was*.”(V12/Ph)

## Discussion

The aim of the overall mixed methods study was to identify and understand the factors affecting the management of an emerging zoonosis: HeV, and the related veterinary IC issues experienced by private veterinary practitioners. The results presented here focused on a subset of the study that examined the HeV-related risk communication challenges between veterinarians and their clients. Most participants from Phases 1 and 2a reported having had access to a HeV management plan and endeavouring to inform their clients about the health and safety risks involved when managing a suspected or confirmed case of HeV. Although, this information was conveyed to horse owners or lay assistants, participants from both phases of the study highlighted a number of communication issues that affected several aspects of HeV management. While some horse owners were receptive to information and safety directives regarding HeV-related risks to animal and human health, others were reportedly unreceptive to these recommendations. Reasons for lack of responsiveness from horse owners included: 1) exposure had already occurred; 2) risk was not properly understood or was denied; 3) scepticism about the risk within the rural culture; 4) cost of IC and/or testing for HeV; 5) fear of long-term detrimental effect to personal or business reputation; and 6) emotional state of clients. Participants in both studies reported that the lack of co-operation from some clients hampered their efforts to follow official guidelines when managing a potential HeV outbreak. Some participants went further by stating that these communication issues prevented them and/or their practice from fulfilling their workplace health and safety and biosecurity legal responsibilities.

Private veterinarians managing early HeV outbreaks were confronted with several challenges including: slow pattern of HeV emergence, overcoming work culture with poor IC standards and misconceptions about zoonotic risks; lack of experience managing emerging zoonoses; lack of professional and governmental leadership; dispatching samples for diagnosis; and managing a biosecurity, public health and occupational health and safety issue in the context of a private business [40, 41, 47–49]. Regardless of these difficulties, veterinarians are required to communicate effectively with their clients about the associated risks to human health and to encourage horse owners to adhere with veterinary risk mitigation advice. Clients’ adherence with veterinary recommendations has been shown to improve when a “client-centred care” approach is used in consultations and this approach is based upon a collaborative interaction between the veterinarian and the client which involves shared decision making to support adherence with veterinary recommendations [27]. Hence, there is a growing impetus to design and provide adequate training for veterinary students and practising veterinarians to improve their communication and relationship building skills with their client [3, 4, 50]. Some of the veterinarians interviewed and surveyed for this study did not seem to experience any difficulty communicating with their clients about HeV risks or ensuring adherence with their IC directives; while others found it difficult. The communication skills of these participants may have been acquired through training and experience and/or been an innate personal quality. Since Phase 1 started before a State-wide government campaign of information about HeV management, some veterinarians may have found it difficult to communicate with their clients because they were insufficiently informed about the related risks or lacked confidence in their knowledge of the risks [51]. Some early career veterinarians have also reported having been misinformed about the risks of HeV because of discrepancies in undergraduate curricula between Australian universities [41]. So, unless veterinarians are adequately informed about a particular zoonotic risk it is likely they will experience some difficulties communicating with clients about the associated risks. Furthermore, it has been suggested that communication about risks related to animal diseases that could affect human health directly (zoonoses) or indirectly (affecting food safety) is not the sole responsibility of veterinarians and should be led by relevant government agencies [52]. In the case of HeV, two independent reports and an Ombudsman’s report about the response of the Qld government to the early HeV outbreaks made similar recommendations [47–49].

When faced with a potential case of HeV, some clients were described as being fearful of the risks, or disbelieving the severity of the risks. This may have been due to a lack of knowledge and understanding about the risks associated with this emerging zoonosis. Kung and colleagues (2013) reported that horse owners in the geographic distribution range of HeV had inconsistent knowledge about the disease. In addition, although more than half recognised HeV was likely to occur in their area, only a third would think of HeV if their horse became ill [15]. Moreover, they also reported inconsistent uptake of the recommendations provided to horse owners in terms of minimising the exposure of horses to HeV [15]. Scheman and colleagues (2011) found that horse owners not directly involved in the horse industry had much lower perception of risk and lower adherence with biosecurity advice during an equine influenza outbreak than horse owners directly involved in the industry [14]. Veterinarians may therefore need to assess a client’s level of prior knowledge about a particular risk, zoonotic or otherwise, in order to tailor the risk communication message.

Research suggests that generally clients perceive veterinarians as the animal health professional equivalent of their own physician [7]. Yet, participants of both Phases 1 and 2a reported that the expert information, advice and assessment of the risk they provided was overlooked or ignored by clients. This seemed to occur more frequently in rural areas where local traditional beliefs prevailed over veterinary knowledge. Australian horse owners from rural areas have also previously been found to overlook preventive veterinary healthcare more often than those from urban areas [12]. This is in contrast with other research which showed that animal producers recognised veterinarians as experts in animal biosecurity; while domestic animal owners did not value veterinary expertise other than when an animal appeared seriously ill [21, 24, 25]. The lack of trust in veterinary expertise may need to be further explored in order to understand its origin and its effect on the veterinary-client relationship. Veterinarians need to be aware of the way in which they and their expert opinion is perceived by clients. It has been suggested that veterinarian-client trust issues could be overcome by veterinarians establishing a relationship built on an inclusive collaboration with clients, which is more likely to yield client cooperation [17, 27]. However, in the case of the management of a lethal emerging disease such as HeV, biosecurity and occupational health and safety take precedent which may affect the dynamics of the client-centred care approach.

In Australia, HeV is a notifiable disease and veterinarians are legally responsible for warning their clients about risks and providing them with occupational health and safety procedures to avoid exposure [28, 35]. Despite holding this responsibility, veterinarians do not have the legal authority to enforce client’s adherence with veterinary health and safety directives [39]. This can leave private veterinarians vulnerable to legal liability. A client experiencing a negative health outcome as a result of non-adherence with veterinary instructions could be interpreted as veterinary malpractice or negligence [39]. However, veterinarians have the legal option to refuse services if they deem a work situation unsafe [53]. The personal, professional and business interests of veterinarians which are vested in having clients adhere with occupational health and safety recommendations may differ from clients’ motivations when seeking veterinary advice. This misalignment of motivations may also be the source of miscommunication. In this study, the attitudes of some clients, as perceived by veterinarians, appeared to be based on: the misperception or misunderstanding of the risks involved; financial and reputation concerns; and/or emotional state of clients. This mismatch between veterinary and client views is not uncommon in veterinary practice [16–20]. For example, some clients tend to focus on the wellbeing of the animal, while veterinarians focus on delivering information about the health status of the animal from a clinical perspective [16–20]. Sayers and colleagues (2014) found that although some farmers viewed veterinarians as the experts, their motivations were very different from those of their veterinarians [21]. While farmers were more motivated by the health of their animals, veterinarians were motivated by cost benefits and legal requirements [21]. When a client did not expect or believe a HeV diagnosis, the cost incurred by the management of HeV was more likely to become an issue that was reported by several participants from Phase1 and 2a. Other cost issues associated with transport of biological samples and diagnostic testing for HeV have been previously reported [7]. In some cases the level of legal responsibility combined with the difficulties in communicating with clients rendered the situation too burdensome for some veterinarians, who decided to cease equine practice [40]. Veterinarians who continue to provide veterinary equine services, may need to recognise that clients have their own motivation and emotional drives. These considerations should be integrated into a client-centred approach to communicating with clients in order to improve the veterinary-client relationship and yield better outcomes, including health and safety, for all.

One underlining element that may have also hampered the communication between veterinarians and horse owners was the topic of conversation: risks surrounding an emerging zoonosis. Emerging zoonoses are unknown and unpredictable events. Prior to 2011, the epidemiology of HeV was not fully elucidated and outbreaks were rare [41]. Suspecting a horse of being infected with HeV represented an unfamiliar scenario for most horse owners and veterinarians. For example, the case definition of HeV was non-specific [8, 41, 54]. It was also later found that infected animals could shed viral particles up to 2 days prior to developing clinical signs, which means the risk could be present but not apparent [37]. The HeV-risks were therefore not easily identifiable. Furthermore, in order to efficiently manage a potential outbreak, a veterinarian would need to not only first suspect HeV, but also to promptly implement an appropriate management plan [35]. Mitigating a severe but ambiguous occupational risk in a short amount of time would be akin to dealing with a crisis situation, which may have affected the interactions between veterinarians and their clients. Telg (2013) differentiates between “risk communication”, about the safety of certain known scenarios and “crisis communication”, which addresses unpredictable risks [26]. The former proactively aims to prevent known unsafe events, while the latter tends to react to an unforeseen situation. The slow and erratic pattern of emergence of HeV was anything but predictable and has been reported to have hindered many aspects of HeV management [41]. The attributes necessary to communicate effectively during a crisis situation may differ from those required during routine interactions; this requires further investigation within the veterinary context. This aspect of veterinary communication is likely to become more relevant as an increasing number of emerging infectious diseases are zoonotic in nature and veterinarians are more likely to be confronted with unpredictable zoonotic events in the future [55, 56]. Veterinarians have to be better prepared to manage low incidence, high-consequence pathogens such as HeV [57].

Since this study was undertaken, some of the issues have been addressed by government agencies through the Intergovernmental Task Force, professional peak bodies and the National Hendra Virus Research Program [58, 59]. For example the HeV management guidelines and recommendations to veterinarians have been widely distributed throughout Qld and the cost of PPE is now also subsidised by the government [38]. Although this has helped improve the management of potential cases of HeV, some veterinarians still fail to comply with biosecurity directives as the recent prosecution of three veterinarians, who failed to protect themselves and/or inform and instruct their client about the risks of exposure to HeV, demonstrated [60]. These prosecutions would have serious implications for the veterinary profession if the veterinarians concerned were to be charged in the absence of any adverse effect of the putative exposure to the virus. These prosecutions may also have implications for the medical and nursing professions because although breaches of IC occur very frequently in hospitals and clinics, they currently do not result in any legal workplace health and safety action, particularly when no harm arises from the lapse [61]. In addition, communication about HeV has become more complicated and controversial as Qld veterinarians refuse to examine horses that have not been vaccinated against HeV [62].

There were several limitations to this study. The results presented here were part of a larger mixed methods study which focused on the perspectives of veterinarians only and not that of their clients. Phase 1 of the study did not specifically set out to examine communication issues, however, once this topic emerged as an important theme it was further explored during Phase 2a. Not all veterinarians who participated in Phase 2a answered the open-ended questions, and the research tool did not permit the clarification of responses of those who answered these questions. This may have introduced a bias towards those who experienced communication difficulties more frequently, and may also be the reason why one of the themes from Phase 1 did not recur during Phase 2a. However, this was not quantified during the survey. Nevertheless, the triangulation of qualitative data from both phases proved valuable in crystallising several factors affecting HeV-related risk communication between veterinarians and their clients. However, in order to fully understand the communication issues between veterinarians and horse owners it would be necessary to also explore the views of the latter.

## Conclusion

Although, some of the communication issues experienced by veterinarians, and reported in these studies, were similar to those experienced during routine veterinary practice, other issues were associated more specifically to the emergence of HeV. When faced with an emerging zoonoses, veterinarians need to be promptly informed of the risks involved to give them the necessary knowledge to assess and explain the risks to their clients. In turn, veterinarians need to acknowledge that in such circumstances their clients are likely to lack the necessary information to appreciate the related risks and that a collaborative approach to communicating is likely to help get the message across. There needs to be further investigation of the particular skills or personal attributes that are necessary to communicate effectively in these kinds of crisis situations, emerging zoonotic outbreaks, within the veterinary context in order to better train existing and future veterinarians. Veterinarians also need to be aware of the ways in which their expertise is perceived and the motivation of their clients, in order to achieve better communication. This could be achieved by implementing a client-centred approach to veterinary communication with the aim of not only improving the veterinarian-client relationship but also achieving positive health and safety outcomes for both veterinary staff and their clients when dealing with zoonotic risks.

## Additional files

Additional file 1: Interview Questionnaire Phase 1. (DOCX 96 kb)
Additional file 2: Survey Questionnaire Phase 2a. (DOCX 492 kb)
