# Supplementary material for: Difficulties experienced by veterinarians when communicating about emerging zoonotic risks with animal owners: the case of Hendra virus
Source: BMC Vet Res. 2017 Feb 18;13:56. doi: 10.1186/s12917-017-0970-2 (PMC5316153; doi:10.1186/s12917-017-0970-2)
Supplement: Supplementary file 1 [file 12917_2017_970_MOESM1_ESM.docx]

JAMES COOK UNIVERSITY
TOWNSVILLE Queensland 4811 Australia Telephone: (07) 4781 4111

INTERVIEW QUESTIONNAIRE

Improving Infection control in Equine Veterinary Practice

Question 1-
When finding out about Hendra virus related deaths of colleagues, what were your immediate thoughts and concerns?

Question 2-
Did it make you modify the way you practice? If yes, in what way?

Question 3-
Who do you contact when seeking professional advice and support on the topic of Hendra virus and infection control in equine vet practice?

- a- People
- b- Organisations

Question 4-
What does the Workplace Health and Safety Act mean to you in relation to your work?

Question 5-
What do you believe are your responsibilities when attending to a horse?

- a- To other staff
- b- To Owners/ Handlers

Question 6-
What are the main obstacles to the use of infection control in equine vet practice?

- a- Cost
- b- Safety
- c- Practicability
- d- Comfort

Question 7-
What do you think will bring change/make a difference to infection control in equine vet practice?

- a- You
- b- Your practice
- c- Vet profession

Question 8-
Is there anything else you would like to add regarding this topic that we might not have addressed through this interview?
