# Supplementary material for: Difficulties experienced by veterinarians when communicating about emerging zoonotic risks with animal owners: the case of Hendra virus
Source: BMC Vet Res. 2017 Feb 18;13:56. doi: 10.1186/s12917-017-0970-2 (PMC5316153; doi:10.1186/s12917-017-0970-2)
Supplement: Supplementary file 2 [file 12917_2017_970_MOESM2_ESM.docx]

Infection control and Hendra virus case management in Queensland equine and mixed veterinary practices

PLEASE ENSURE THAT YOU READ AND ANSWER ALL THE QUESTIONS ON THIS FORM BY TICKING THE APPROPRIATE BOX ✓
(multiple answers are ok for most questions).
THANK YOU VERY MUCH FOR YOUR COOPERATION IN THIS IMPORTANT STUDY TO IMPROVE INFECTION CONTROL IN THE PROFESSION.

YOUR REPLY IS VERY IMPORTANT TO US

Gender ☐ Female ☐ Male | **Age** ☐ <30 ☐ 31-40 ☐ 41-50 ☐ >50 | **Post Code:**......................

Veterinary Degree
Country [If not from an Australian University]:................................
University:....................................
Year of graduation:......................

Other Qualifications	Country	Year Awarded

Type of Veterinary work
☐ Clinical practice ☐ Non-clinical

Practice type
☐ Large animals ☐ Mixed ☐ Specialist, please specify:...

Work Status
☐ Part time ☐ Full time ☐ Locum ☐Student ☐ Retired

Average number of hours worked per week
....

Role in practice
☐ Principal ☐ Partner ☐ Employee ☐ Placement

Number of vets currently employed in your practice
Full time................ Part time................

Are you a member of any of the following? ☐AVA ☐EVA
☐Other:...

Frequency with which you provide veterinary services to horses
☐ Daily ☐ Weekly ☐ Monthly ☐ Yearly ☐ Only on call/after hours ☐ Never
☐ Have ceased to provide Veterinary services to horses, if so when and why?...

Type of equine premises you mostly visit
☐ Hobby farm(paddock) ☐ Pony Club/Riding School ☐ Equine Artificial Insemination Centre ☐ All of the above
☐ Farm(paddock) ☐ Performance horse stables ☐ Stud

Do you wear Personal Protective Equipment [PPE] when examining a horse?

**If animal is healthy**	☐ Always	☐ Sometimes	☐ Rarely	☐ Never
**If animal is sick**	☐ Always	☐ Sometimes	☐ Rarely	☐ Never
**If animal is dead**	☐ Always	☐ Sometimes	☐ Rarely	☐ Never
**If carrying out a necropsy**	☐ Always	☐ Sometimes	☐ Rarely	☐ Never

What kind of PPE do you wear when examining a horse [Tick all that applies]?

	If horse is healthy	If horse is sick	If horse is dead	If carrying out a necropsy	If HeV is suspected on a live horse
**Overalls**	☐	☐	☐	☐	☐
**Hat**	☐	☐	☐	☐	☐
**Gown**	☐	☐	☐	☐	☐
**Gumboots/Boot covers**	☐	☐	☐	☐	☐
**Surgical mask**	☐	☐	☐	☐	☐
**Surgical mask with fluid barrier**	☐	☐	☐	☐	☐
**Surgical mask with eye shield**	☐	☐	☐	☐	☐
**Particulate mask (N95/P2)**	☐	☐	☐	☐	☐
**Face shield**	☐	☐	☐	☐	☐
**Powered air respirator**	☐	☐	☐	☐	☐
**Goggles**	☐	☐	☐	☐	☐
**Hand gloves**	☐	☐	☐	☐	☐
**Arm length gloves**	☐	☐	☐	☐	☐
**Apron**	☐	☐	☐	☐	☐
**Protective dressings on your skin abrasions**	☐	☐	☐	☐	☐

Other: ....
....
....
....
....

Please return this questionnaire to:
Rick Speare or Diana Mendez
SPHTMRS James Cook University Townsville QLD 4811

Or fax to:
Rick Speare or Diana Mendez
47961767

If asked, would you do a necropsy on a horse that died of the following causes?

☐ Sickness ☐ Accident ☐ Sudden unexplained death

If not, why?..

....

What are the most common veterinary procedures you conduct on horses?

☐ Dentistry procedure ☐ Trephination of sinuses ☐ Retained foetal membranes
☐ Endoscopy of URT ☐ Gaseous anaesthesia ☐ Abortion/dead foal
☐ Endotracheal intubation ☐ Endoscopy of urinary tract ☐ Necropsy
☐ Thermocautery of soft palate ☐ Catheterisation of the bladder ☐ Wound management
☐ Sampling for pharyngeal biopsy ☐ Artificial insemination ☐ All of the above
☐ Injection of ethmoid haematomas ☐ Foaling

Have you ever considered refusing to attend to a horse for any of the following reasons?

**Hendra risk to human health**	☐ Always	☐ More than once	☐ Once	☐ Never
**Litigation risk associated with Hendra**	☐ Always	☐ More than once	☐ Once	☐ Never
**Hendra risk related added cost/time**	☐ Always	☐ More than once	☐ Once	☐ Never

How would you rate the level of Hendra virus [HeV] related risk to human health when performing the following veterinary procedures on a horse?

**Clinical examination of a healthy horse**	☐ Very high	☐ High	☐ Moderate	☐ Low	☐ Nil
**Clinical examination of a horse displaying respiratory symptoms**	☐ Very high	☐ High	☐ Moderate	☐ Low	☐ Nil
**Clinical examination of a febrile horse**	☐ Very high	☐ High	☐ Moderate	☐ Low	☐ Nil
**Clinical examination of a horse displaying neurological symptoms**	☐ Very high	☐ High	☐ Moderate	☐ Low	☐ Nil
**Clinical examination of a sick horse displaying symptoms other than the ones mentioned above**	☐ Very high	☐ High	☐ Moderate	☐ Low	☐ Nil
**Equine dentistry**	☐ Very high	☐ High	☐ Moderate	☐ Low	☐ Nil
**Wound management**	☐ Very high	☐ High	☐ Moderate	☐ Low	☐ Nil
**Endoscopy**	☐ Very high	☐ High	☐ Moderate	☐ Low	☐ Nil
**Foaling**	☐ Very high	☐ High	☐ Moderate	☐ Low	☐ Nil
**Artificial insemination**	☐ Very high	☐ High	☐ Moderate	☐ Low	☐ Nil
**Necropsy**	☐ Very high	☐ High	☐ Moderate	☐ Low	☐ Nil

How would you rate the level of HeV related risk to human health when exposed to the following equine bodily fluids?

**Sweat**	☐ Very high	☐ High	☐ Moderate	☐ Low	☐ Nil
**Blood**	☐ Very high	☐ High	☐ Moderate	☐ Low	☐ Nil
**Nasal secretions**	☐ Very high	☐ High	☐ Moderate	☐ Low	☐ Nil
**Sputum**	☐ Very high	☐ High	☐ Moderate	☐ Low	☐ Nil
**Saliva**	☐ Very high	☐ High	☐ Moderate	☐ Low	☐ Nil
**Foetal material**	☐ Very high	☐ High	☐ Moderate	☐ Low	☐ Nil
**Reproductive tract secretions**	☐ Very high	☐ High	☐ Moderate	☐ Low	☐ Nil
**Urine**	☐ Very high	☐ High	☐ Moderate	☐ Low	☐ Nil
**Manure**	☐ Very high	☐ High	☐ Moderate	☐ Low	☐ Nil
**Any of the above in the form of visible droplets**	☐ Very high	☐ High	☐ Moderate	☐ Low	☐ Nil
**Any of the above in the form of aerosolised droplets**	☐ Very high	☐ High	☐ Moderate	☐ Low	☐ Nil

In your opinion what are the main barriers to the use of PPE in the field when examining a horse?

☐ Heat stress ☐ Finding the time to source adequate PPE ☐ Reaction of horse to altered appearance
☐ Facial hair ☐ Time & cost to learn to use specialised PPE ☐ Negative perception from the owner
☐ Practicability ☐ Added cost due to infection control [IC] ☐ Negative perception from non-PPE user colleagues
☐ Comfort ☐ Time & cost to maintain specialised PPE
☐ Gauging when PPE needed ☐ Increased time of consultation due to IC
☐ Other, please specify:..

....

....

How could some PPE you use be improved?

....

....

....

Does your practice have a HeV management response plan? ☐ Yes ☐ No ☐Don't know

Does your HeV management response plan include?

☐ A copy of official set of guidelines for veterinarians handling horses potentially infected with HeV.
If yes, please specify source of guidelines:...

☐ Set of specific policies/standard procedures
☐ Risk assessment management plan
☐ Phone triage system
☐ Fact sheet about Hendra virus for horse owners
☐ Fact sheet about flying foxes for horse owners
☐ Dedicated field kit (with appropriate PPE)
☐ Record of field kit maintenance
☐ Record of specific staff training (IC & PPE)
☐ Case reporting system
☐ Occupational incident reporting system

Type of HeV field kit used in your practice

☐ Don't have one
☐ Kit put together in house
☐ Commercial kit, please specify brand:
....

Do you think your HeV kit is complete?

☐ Yes ☐ No ☐ Never used it ☐ Don't have one

If no, how could it be improved?

....

....

What does your HeV field kit include?

☐ Don't have one
☐ Never looked at it
☐ Surgical masks [SM]
☐ SM with fluid barrier
☐ SM with eye shield
☐ Face masks
☐ Powered air respirator
☐ Goggles
☐ Hat
☐ Gumboots/ Boot covers
☐ Reusable overalls
☐ Disposable overalls
☐ Disposable gloves
☐ Cut resistant glove liners
☐ Change of clothing
☐ Diagnostic sample equipment
☐ Diagnostic sample packaging
☐ Portable sharps disposable unit
☐ Protective dressing for skin abrasions
☐ Biological disposable bags
☐ Hand disinfecting solution
☐ Equipment disinfecting solution
☐ Other, please specify: ....
....

How often do you check the content of your HeV field kit for maintenance?

☐ Weekly ☐ Monthly ☐6 monthly ☐ Yearly ☐ Never if not used ☐ Don't have one

Does your practice have an isolation stable or yard? ☐ Yes ☐ No ☐ Don't know

How would you rate your experience with diagnostic testing for HeV?

☐ Excellent ☐ Good ☐ Difficult ☐ Poor

Why?

☐ Technical sampling difficulties
☐ Finding a courier to dispatch samples
☐ Finding a testing laboratory
☐ Acceptance of cost by client
☐ Getting timely results
☐ Other tests put on hold until HeV is excluded
☐ Other, please specify: ....

....

....

Do you educate your clients on the risks and management of potential cases of HeV?

**If animal is healthy**	☐ Always	☐ Sometimes	☐ Rarely	☐ Never
**If animal is sick**	☐ Always	☐ Sometimes	☐ Rarely	☐ Never
**If animal is dead**	☐ Always	☐ Sometimes	☐ Rarely	☐ Never
**If assisting with a horse necropsy**	☐ Always	☐ Sometimes	☐ Rarely	☐ Never

When do you instruct lay assistants on their risk of potential exposure to HeV?

**If animal is healthy**	☐ Always	☐ Sometimes	☐ Rarely	☐ Never
**If animal is sick**	☐ Always	☐ Sometimes	☐ Rarely	☐ Never
**If animal is dead**	☐ Always	☐ Sometimes	☐ Rarely	☐ Never
**If assisting with a horse necropsy**	☐ Always	☐ Sometimes	☐ Rarely	☐ Never

What is their reaction upon receiving such instructions?

....

....

....

When do you ask lay assistants to use PPE to minimise their risk of potential exposure to HeV?

**If animal is healthy**	☐ Always	☐ Sometimes	☐ Rarely	☐ Never
**If animal is sick**	☐ Always	☐ Sometimes	☐ Rarely	☐ Never
**If animal is dead**	☐ Always	☐ Sometimes	☐ Rarely	☐ Never
**If assisting with a horse necropsy**	☐ Always	☐ Sometimes	☐ Rarely	☐ Never

What is their reaction upon receiving such instructions?

....

....

Does your practice have the following facilities in the area where equine patients are examined?

☐ Hand washing dedicated station
☐ Equipment washing dedicated station
☐ A food & beverage exclusion zone
☐ Separate fridges for food & drugs/diagnostic specimen
☐ Quarantine isolation stable
☐ Biological waste disposal unit(s)
☐ Sharps disposal unit(s)
☐ Sterilisation station [Autoclave]

Have you attended any training session about the risks and management of HeV in the past 12 months? ☐ Yes ☐ No

If yes, please specify training provider and numbers of training hours:..

Have you ever had to deal with a potential case of HeV? ☐ Yes ☐ No

If yes how would you rate the following aspects of this experience

Client response	☐ Excellent	☐ Good	☐ Difficult	☐ Poor	
Financial aspect	☐ Excellent	☐ Good	☐ Difficult	☐ Poor	
Support from relevant authorities	☐ Excellent	☐ Good	☐ Difficult	☐ Poor	
Interfacing with testing laboratories	☐ Excellent	☐ Good	☐ Difficult	☐ Poor	
Staff response	☐ Excellent	☐ Good	☐ Difficult	☐ Poor	
Media response	☐ Excellent	☐ Good	☐ Difficult	☐ Poor	☐ Not involved

Additional comment:..

....

....

....

....

If a HeV vaccine for horses were available, would you?

☐ Still consider HeV a high risk for equine practice
☐ Require all horses you examine to be vaccinated
☐ Return to equine practice if you had stopped because of HeV
☐ No longer consider HeV a high risk for equine practice
☐ Terminate your HeV risk management plan & procedures
☐ Be less concerned about implementing IC

In your opinion, how can infection control in equine veterinary practice be improved?

In the short term:...

....

....

....

....

....

In the long term:...

....

....

....

....

....

Are there any other comments you wish to add on the subject of infection control in equine veterinary practice?

....

....

....

....

Thanks for your time!
